# Occult Grade 5 Splenic Injury in a Haemodynamically Stable Child Following Bicycle Handlebar Injury: A Case Report

**DOI:** 10.7759/cureus.99632

**Published:** 2025-12-19

**Authors:** Ahmed Amer, Gerard Zed Ayaay, Michael Shakhloul, Ahmad El Marakby, Ibrahim Abouelkhir

**Affiliations:** 1 Emergency Department, Royal Surrey County Hospital, Guildford, GBR; 2 Emergency Department, Egyptian Medical Syndicate, Cairo, EGY; 3 Emergency Department, Frimly Park Hospital, Frimley, GBR

**Keywords:** fast scan, handlebar injury, pocus (point-of-care ultrasound), trauma imaging, traumatic splenic injury

## Abstract

Bicycle handlebar injuries are one of the most common mechanisms of major paediatric abdominal trauma, with the spleen being one of the most frequently affected solid organs. Grade 5 lesions represent the most severe and comparatively uncommon subtype. Despite the severity of these injuries, children often present with deceptively minimal abdominal signs and stable vital signs due to robust physiological compensatory mechanisms, making early diagnosis challenging. We describe a 13-year-old boy who sustained a grade 5 splenic injury following a bicycle handlebar impact. He presented as haemodynamically stable with left upper quadrant tenderness, no visible abdominal bruising, and minor facial abrasions. Imaging revealed a shattered spleen with haemoperitoneum and grade 5 splenic laceration. He underwent successful non-operative management and was discharged following an uncomplicated recovery. This case highlights the importance of maintaining a low threshold for imaging in paediatric trauma involving handlebar injuries. Abdominal tenderness in a conscious child following handlebar injury should prompt consideration of abdominopelvic CT, regardless of apparent clinical stability, in accordance with the Royal College of Radiologists Major Paediatric Trauma Radiology Guidance 2024, which recommends early imaging in such high-risk presentations.

## Introduction

Cycling injuries are the third most common mechanism of trauma in children aged 7-13 years, and bicycle-related abdominal trauma has remained among the leading causes of paediatric abdominal injury for over 60 years. Approximately 15% of child cyclist injuries involve handlebar impact, with two-thirds of these resulting in abdominal injury. Handlebar impact is now recognised as the most common mechanism of major paediatric abdominal trauma [1].

The spleen is a highly vascular organ in the left upper quadrant, protected by the lower ribs but vulnerable to blunt impact due to its position and friable parenchyma, particularly in handlebar injuries. Traumatic splenic injuries are classified using the American Association for the Surgery of Trauma Organ Injury Scale (2018 revision), which ranges from grade 1 (subcapsular haematoma <10% surface area, parenchymal laceration <1 cm depth or capsular tear) to grade 5 (shattered spleen, hilar vascular injury with devascularisation, or any injury with splenic vascular injury and active bleeding extending into the peritoneum) [2]. This case describes a haemodynamically stable 13-year-old boy with a grade 5 splenic injury following bicycle handlebar impact, highlighting the deceptive nature of handlebar trauma in children and the critical importance of maintaining a low threshold for early CT imaging in accordance with national guidelines.

This case highlights several critical clinical principles. First, high-grade splenic injuries are rare, with grade 5 injuries accounting for only ~7.7% of isolated paediatric splenic injuries [3]. Second, the deceptive nature of this child's haemodynamic stability, with no signs of an acute abdomen despite a grade 5 laceration, underscores the need to adhere to guideline-recommended thresholds for early CT abdomen-pelvis with contrast in any child presenting with abdominal pain in the context of handlebar injury [4]. Third, these rare and most severe splenic injuries in children can be managed successfully with non-operative strategies when recognised promptly and treated within appropriately resourced paediatric centres.

## Case presentation

A 13-year-old boy was brought to the emergency department in June 2025 after a bicycle accident. While riding down a ramp at approximately 20 mph (32 km/h), he suddenly applied the brakes, causing him to fall forward. He was not wearing a helmet and struck his left upper abdomen on the handlebars before landing on the ground. He sat for approximately ten minutes before standing and was unable to recall events during that period. Since the incident, he has had four episodes of vomiting, pain under the chin, and pain in the left lower chest and left upper quadrant extending to the lower quadrant. He had no past medical history, was on no regular medication, and had no known drug allergies.

On the primary survey, the airway was patent, and the cervical spine was clinically evident, with no tenderness and a full range of movement. Breathing was normal, with equal bilateral air entry and no respiratory distress. The chest was clear on auscultation, with tenderness over the left lower ribs. Oxygen saturation was 98% on room air. Circulation was haemodynamically stable, with BP 115/70 mmHg, HR 86 bpm, warm peripheries, normal heart sounds, and good peripheral perfusion. The abdomen was soft, with mild tenderness over the left lower chest and left upper quadrant, with no signs of peritonism or acute abdomen. The pelvis was stable, with no signs of shortening, rotation, or features suggestive of pelvic fracture. Neurological assessment revealed a GCS of 15, equal and reactive pupils, no focal neurological deficits, good symmetrical motor strength in all four limbs, and intact sensation.

The secondary survey demonstrated abrasions over the forehead and chin. The ear, nose, and throat examination was unremarkable. Ocular and periorbital structures were intact. There was infraorbital tenderness with an abrasion on the left side of the face. The oral cavity was unremarkable, with good dental alignment and a jaw opening greater than three fingerbreadths. The pelvis was non-tender with no deformity. The lower limbs were normal with no abnormalities detected. The upper limbs showed abrasions on the left hand with a normal range of movement and no deformities. Log roll and back examination revealed no spinal tenderness.

A decision was made to perform a CT trauma scan comprising a non-contrast CT of the head and cervical spine and a contrast-enhanced CT of the thorax, abdomen, and pelvis. This was undertaken due to the history of vomiting, clinical features of head injury, and suspicion of a handlebar injury.

**Figure 1:**
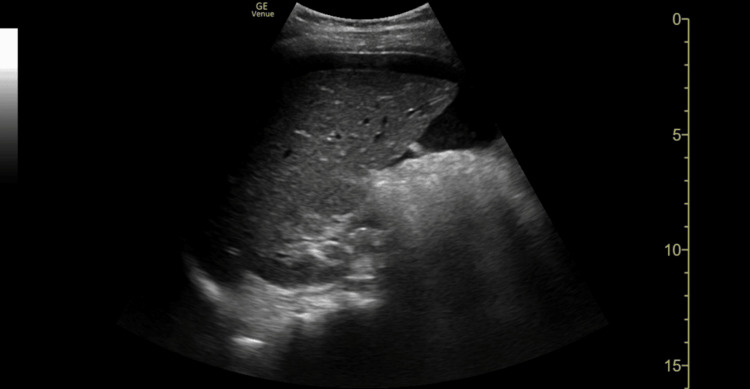
FAST scan of the RUQ demonstrating free intraperitoneal fluid at the caudal tip of the liver FAST: focused abdominal sonography in trauma, RUQ: right upper quadrant

**Figure 2:**
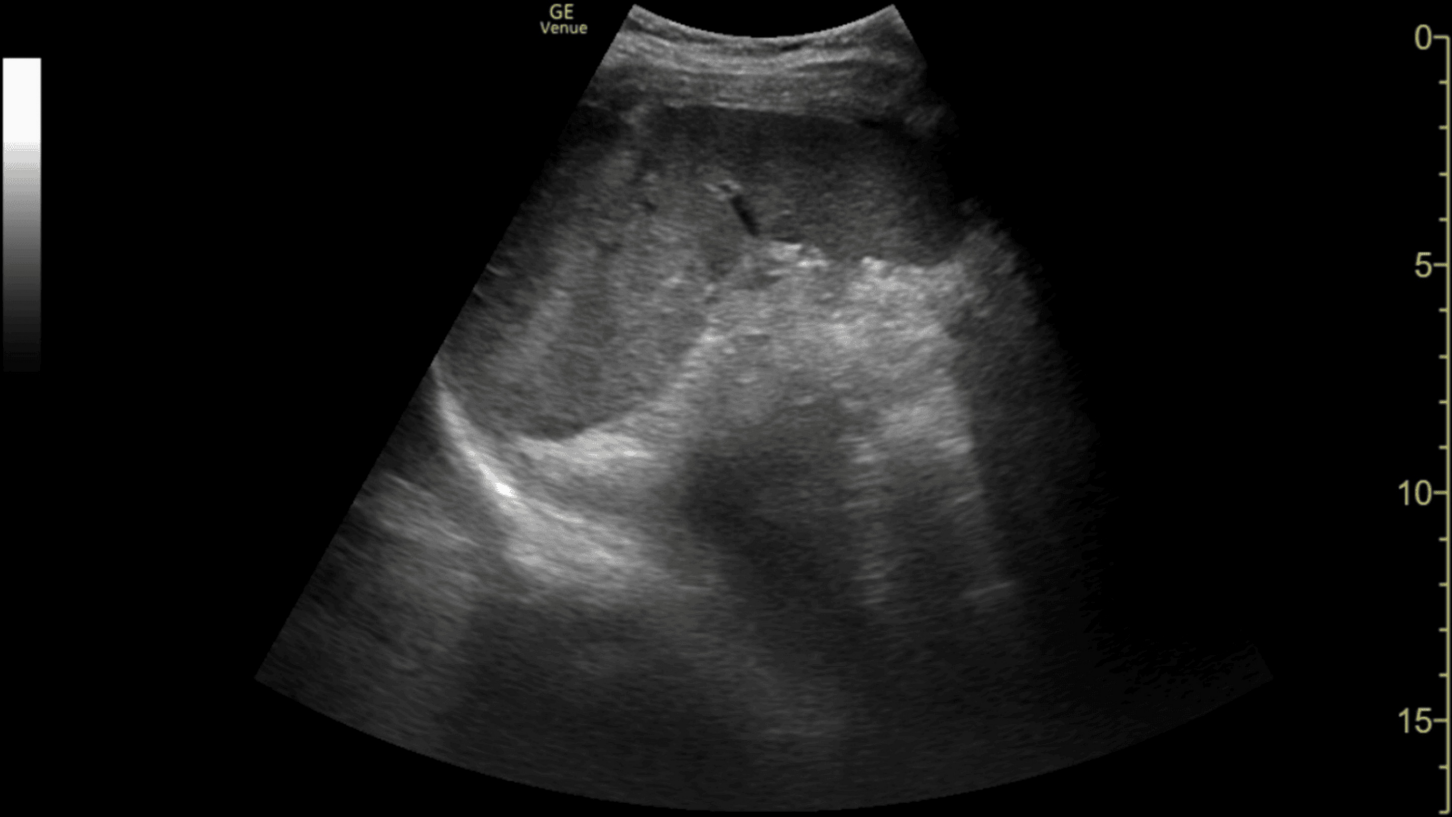
FAST scan of the left upper quadrant showing a normal appearance with no free fluid between the spleen and left kidney FAST: focused abdominal sonography in trauma

**Figure 3:**
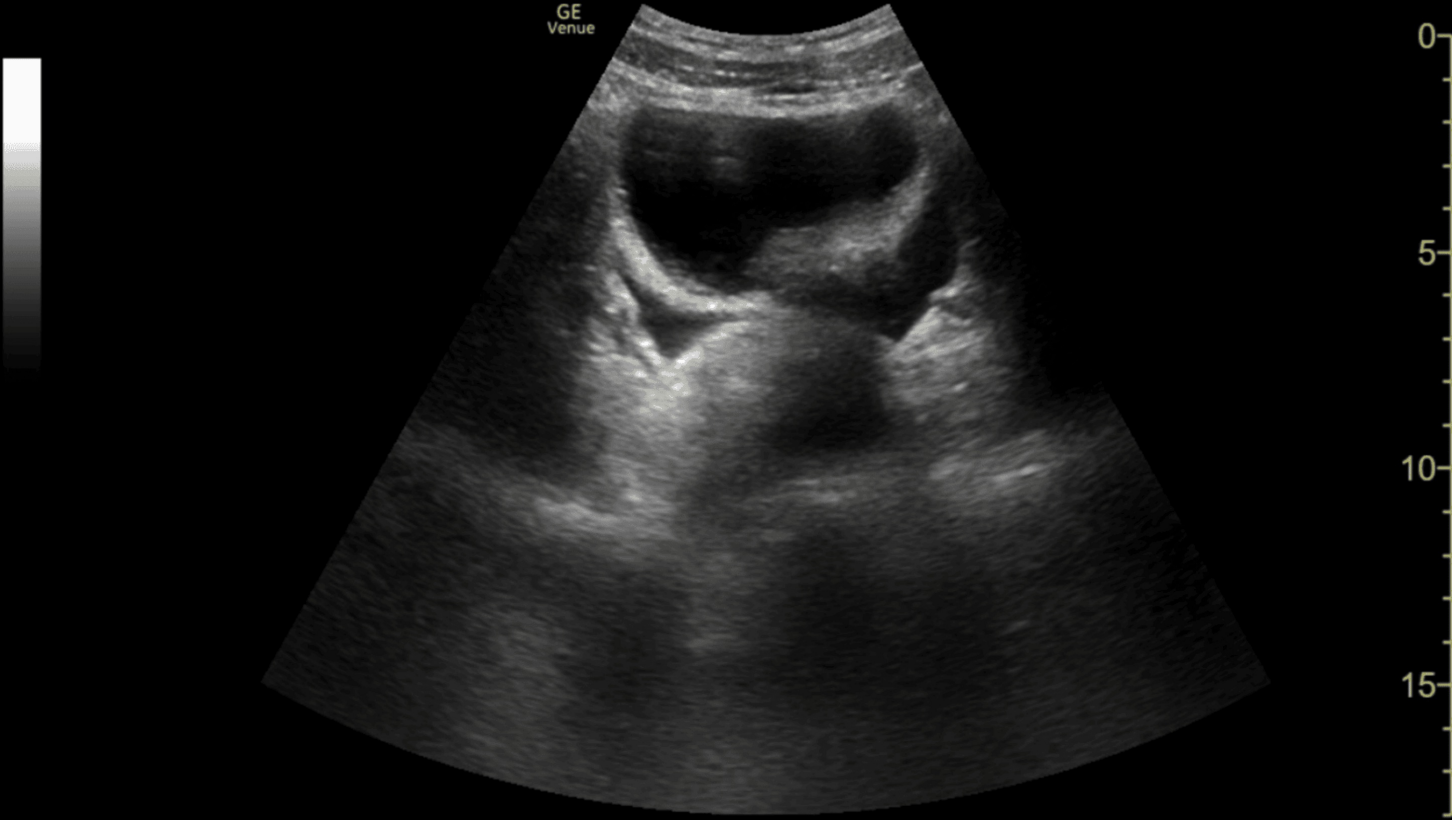
FAST scan, suprapubic transverse view, demonstrating free fluid in the pelvic space FAST: focused abdominal sonography in trauma

**Figure 4:**
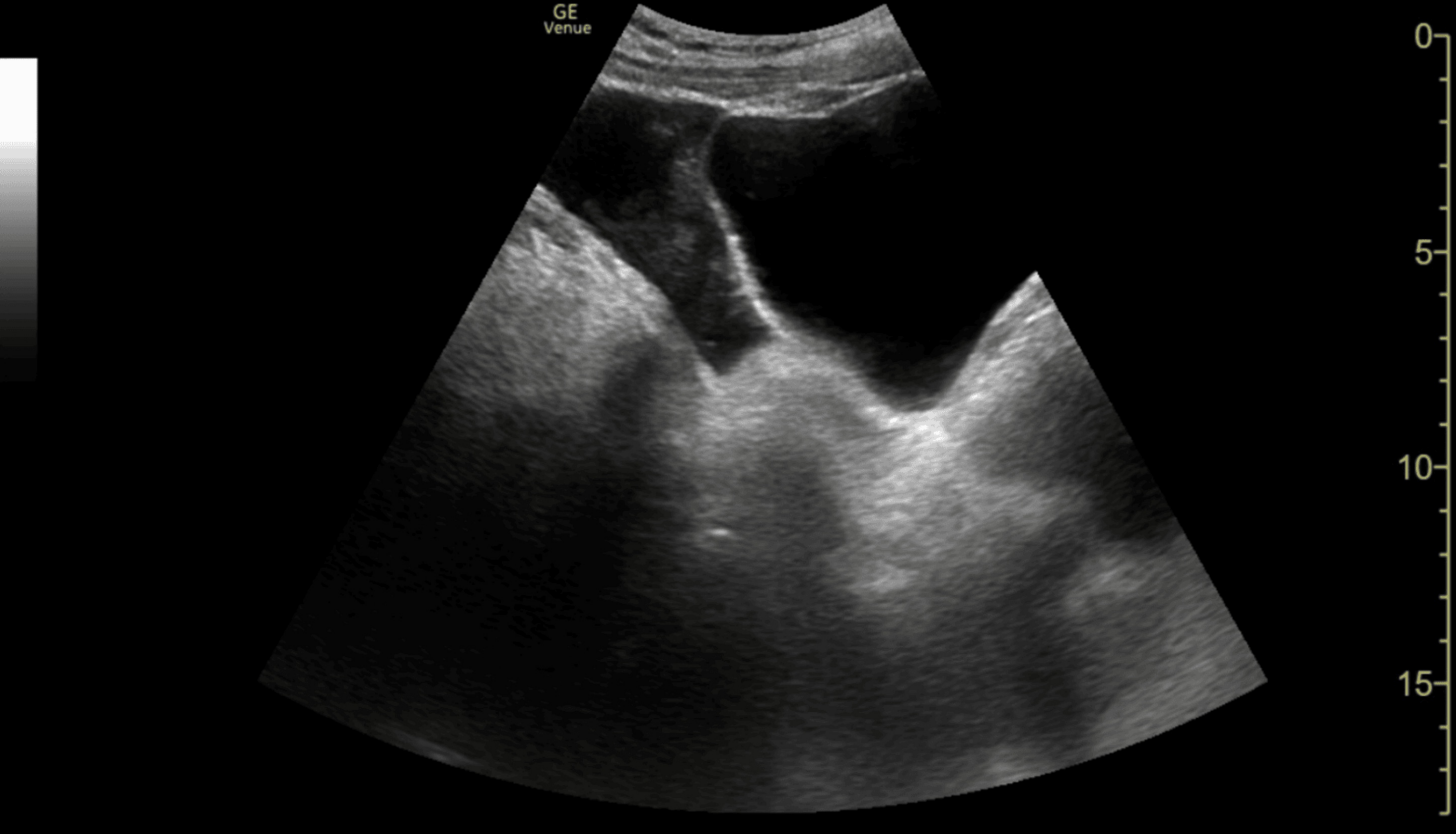
FAST scan, suprapubic longitudinal view, demonstrating free fluid collecting posterior to the bladder FAST: focused abdominal sonography in trauma

**Figure 5:**
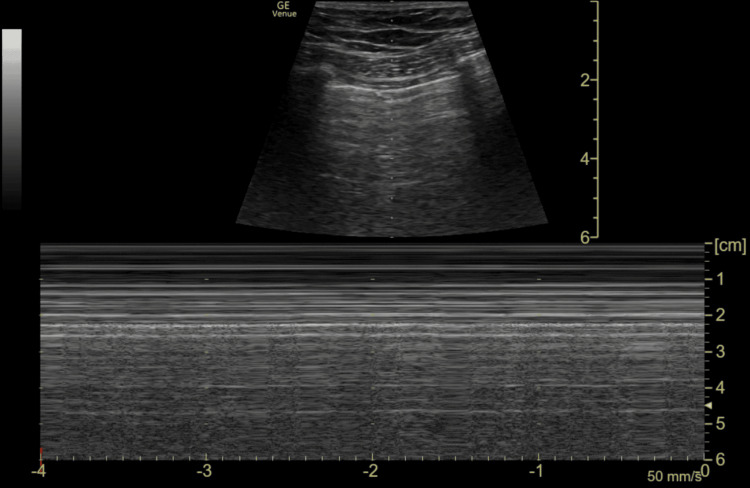
Lung ultrasound of the right lung. B-mode and M-mode images demonstrate normal lung sliding with a seashore sign, excluding pneumothorax

**Figure 6:**
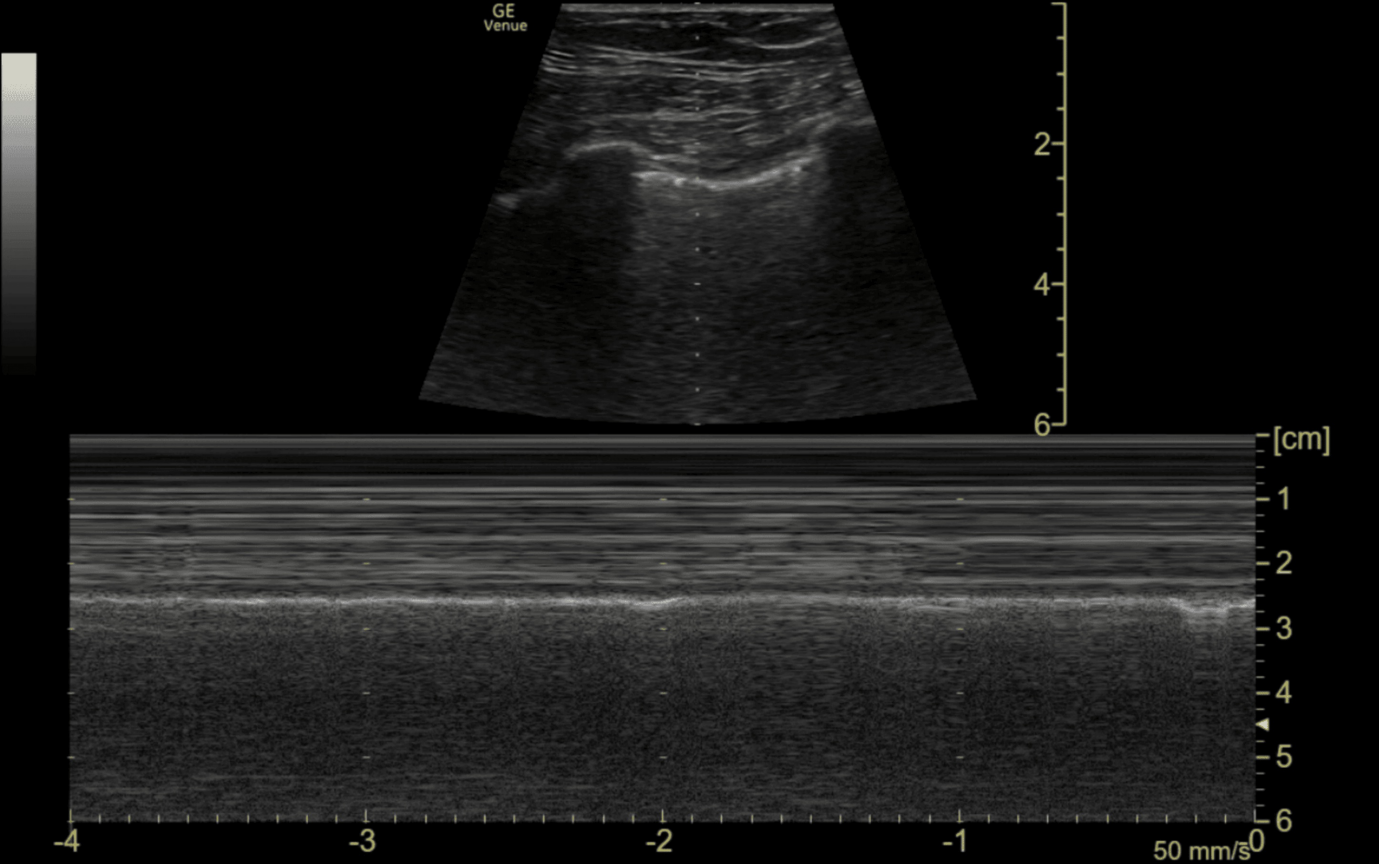
Lung ultrasound of the left lung. B-mode and M-mode images demonstrate normal lung sliding with a seashore sign, excluding pneumothorax

A bedside focused abdominal sonography in trauma (FAST) was performed. In this case, the FAST demonstrated free fluid in Morison's pouch and in the suprapubic transverse and longitudinal views, findings suggestive of haemoperitoneum (Figures 1-6).

**Figure 7:**
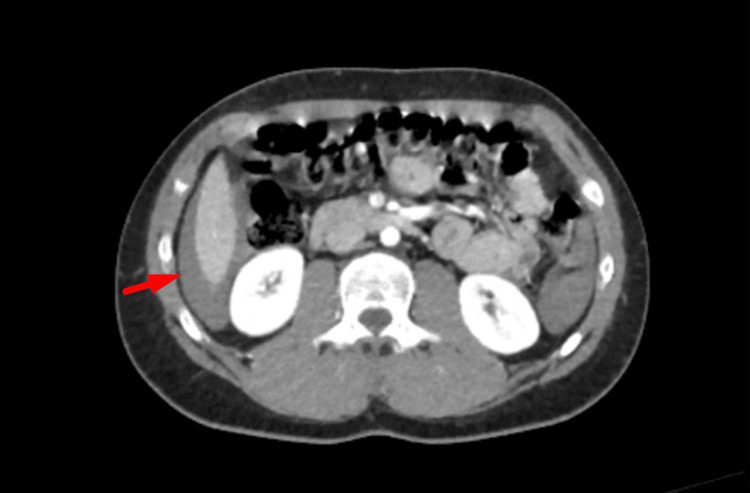
CT cross-section demonstrating haemoperitoneum CT: computed tomography

**Figure 8:**
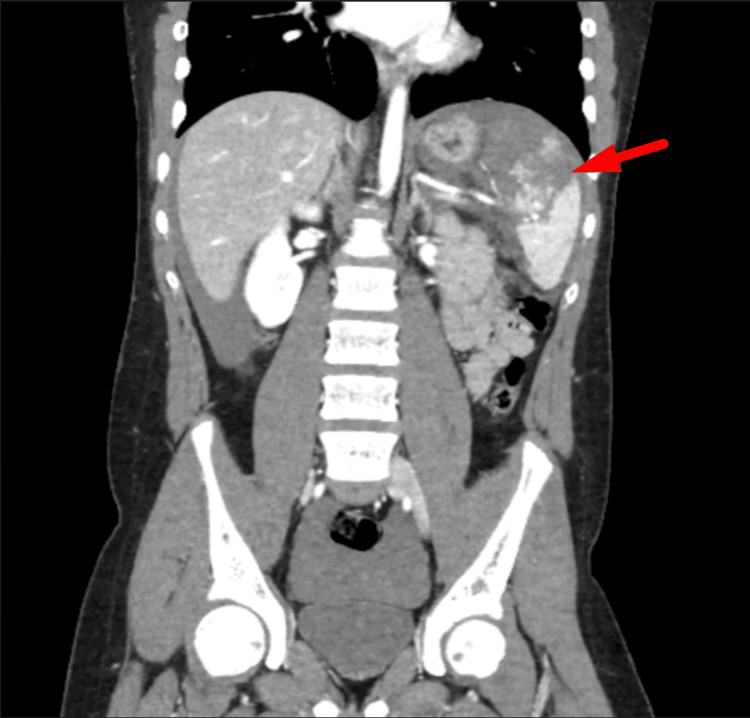
CT axial view demonstrating a grade 5 splenic laceration CT: computed tomography

**Figure 9:**
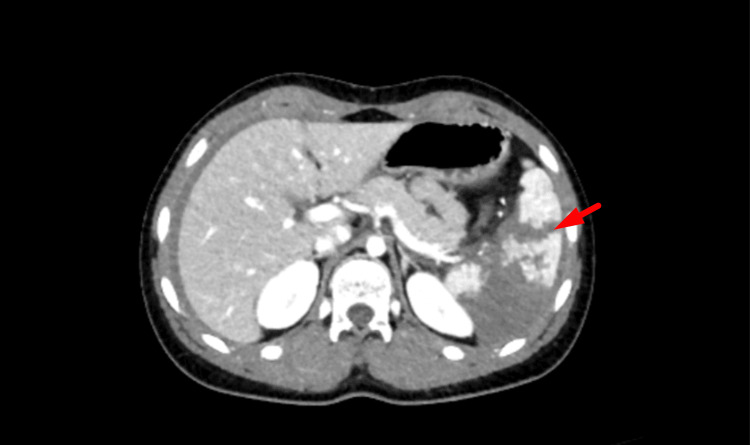
CT cross-section showing rounded intraparenchymal foci consistent with splenic pseudoaneurysms CT: computed tomography

Subsequent imaging was performed to evaluate the extent of injury. CT of the head and cervical spine was unremarkable, showing no acute intracranial abnormality. A contrast-enhanced CT of the thorax, abdomen, and pelvis demonstrated an extensive grade 5 splenic laceration involving more than 50% of the splenic parenchyma, consistent with a shattered spleen. There was significant haemoperitoneum extending throughout the abdomen and pelvis, and multiple rounded foci within the splenic parenchyma were suggestive of pseudoaneurysms. No active contrast extravasation or other visceral or bony injuries were identified (Figures 7-9).

The patient was transferred to a regional trauma centre for ongoing non-operative management (NOM) of his grade 5 splenic injury. He was admitted to the paediatric intensive care unit for two nights of close haemodynamic monitoring. His haemoglobin remained stable (>118 g/L) throughout admission, and he made an uncomplicated recovery.

He was discharged with clear safety-netting advice, including instructions to return if abdominal pain worsened or if any new concerns developed. He was advised to remain out of school until the end of the term, avoid physical activity for seven weeks, and refrain from cycling or high-impact activities for at least three months. No routine follow-up was required.

## Discussion

The patient is a 13-year-old boy who presented with abdominal pain and vomiting following a handlebar impact, representing the typical demographic and clinical profile described in the literature. Handlebar injuries occur most frequently in school-aged boys and characteristically involve the upper abdomen. In the largest systematic review of paediatric thoracoabdominal handlebar trauma, Cheung et al. analysed 1,072 children (mean age 9.7 years; 85.1% male) and identified abdominal pain (54.7%), vomiting (22.4%), and a circular handlebar imprint in approximately one in five cases as predominant presenting features [5]. Notably, our patient exhibited no visible bruising at the impact site despite having sustained a grade 5 splenic laceration, demonstrating that blunt abdominal trauma in children can cause significant internal organ injury despite subtle or absent external signs. Whilst the liver and pancreas have been reported as the most frequently injured organs following handlebar trauma, splenic injuries are well documented [5-8]. High-grade splenic injuries remain comparatively uncommon, comprising approximately 27% of isolated paediatric splenic injuries in UK practice, with grade 5 accounting for only 7.7% [3].

The haemodynamic stability observed in our patient despite a grade 5 splenic injury merits particular discussion. Paediatric patients can sustain substantial blood loss yet maintain normal blood pressure by increasing systemic vascular resistance and preserving cardiac output. This finding is consistent with the well-described paediatric physiological reserve that enables maintenance of blood pressure until approximately 30-40% of circulating blood volume is lost. Hypotension is therefore a late and ominous sign in children [9].

Notably, the Cheung et al. systematic review found that clinical features suggestive of physiological compromise were rare: tachycardia was reported in 0.7% of cases, and pallor in 3.0%, and cardiac arrest/cardiogenic shock/bradycardia occurred in 0.6% of reported examination findings [5]. Our patient has presented similarly, maintaining entirely normal vital signs (BP 115/70 mmHg, HR 86 bpm) and stable haemoglobin levels (>118 g/L) throughout admission despite harbouring a grade 5 splenic laceration with extensive haemoperitoneum.

Handlebar injuries were found to be ten times more likely to result in severe injury. However, more than half of affected children were misdiagnosed at their initial presentation, with delayed diagnosis and longer hospital stays observed in those with abdominal involvement [10].

Consequently, the primary clinical question must always be, "Does this child need a FAST scan at all?" rather than defaulting to comprehensive cross-sectional imaging. FAST scan remains a valuable adjunct in trauma assessment but has recognised limitations. The caudal edge of the liver in the right upper quadrant (RUQ) view is the most sensitive area for detecting free fluid [11]. In this case, FAST demonstrated free fluid at the caudal edge of the liver in the RUQ view, as well as in the suprapubic transverse and longitudinal views, consistent with haemoperitoneum.

While FAST can rapidly detect free fluid, it may not be suitable for assessing blunt abdominal trauma in paediatrics and could provide false reassurance [4]. A normal FAST scan cannot reliably exclude intra-abdominal injury [12]. A positive FAST, as seen in this patient, provides strong justification for subsequent CT imaging. In contrast, a negative FAST scan does not reliably exclude intra-abdominal injury, and further imaging should be guided by clinical suspicion and mechanism of injury. However, unlike in adults, where FAST is a useful decision-making tool, the Royal College of Radiologists does not recommend FAST in paediatric abdominal trauma [4].

Several large systematic reviews have confirmed that while FAST has high specificity, its sensitivity in children is limited. A 2007 meta-analysis including 3,838 children reported a sensitivity of 80% and specificity of 96%, which fell to 66% when lower-quality studies were excluded [13], and a more recent systematic review of 2,135 paediatric patients reported a pooled sensitivity of just 35% [14]. These findings underscore that a negative FAST offers false reassurance and must not be used to exclude injury or defer necessary CT imaging. In contrast, a positive result should prompt further investigation.

The 2024 Major Paediatric Trauma Radiology guidelines provide clear recommendations for imaging. It states: "Where clinically indicated, contrast-enhanced CT of the abdomen and pelvis is the modality of choice for the assessment of acute traumatic intra-abdominal injury" [4]. The Royal College of Radiologists advocates strict adherence to the ALARP principle ("as low as reasonably practicable") regarding ionising radiation in children. It further advises that "single-volume dual-contrast CT is advised to minimise radiation burden" [4]. Notably, the guidance emphasises that the presence of abdominal tenderness in a conscious patient, along with a handlebar injury, should prompt consideration of abdominopelvic CT, even in the absence of haemodynamic instability [4]. This patient met both of these criteria, justifying the decision to proceed with CT imaging.

This case illustrates that severe splenic injury can occur even in the absence of shock or significant signs of external trauma, reinforcing guideline recommendations for a low threshold to perform CT in children with handlebar trauma and abdominal tenderness.

NOM of paediatric blunt splenic injury is associated with high success rates across all injury grades. A 12-year Scandinavian paediatric series reported NOM success rates of 88% without splenic artery embolisation and 94% with embolisation, with no children undergoing immediate splenectomy, supporting the safety of an initial conservative approach even in higher-grade injuries [15]. This is consistent with UK Trauma Audit and Research Network data, which demonstrate that 90.5% of grade 5 splenic injuries in specialist paediatric centres were managed non-operatively, compared with only 56.5% in adult centres [3].

## Conclusions

This case delivers a clear message: bicycle handlebar trauma in children can cause catastrophic internal injury while presenting with deceptively benign clinical findings. Handlebar injuries are among the most common mechanisms of major paediatric abdominal trauma, yet more than half of affected children are misdiagnosed at initial presentation. Our patient sustained a grade 5 splenic laceration, the most severe and uncommon, yet displayed no signs of an acute abdomen, no external bruising, and entirely normal vital signs. Such presentations exploit the paediatric physiological reserve, which maintains haemodynamic stability despite significant intra-abdominal injuries. Clinicians should therefore maintain a low threshold for suspecting occult injury, and any child with abdominal tenderness following handlebar impact warrants early CT imaging in adherence to the 2024 Royal College of Radiologists Major Paediatric Trauma Radiology Guidance, irrespective of reassuring observations or a negative FAST. Early diagnosis not only prevents missed injury but also preserves the opportunity for NOM, which achieves success rates exceeding 90% even for grade 5 injuries when delivered within specialist paediatric centres.
